# An organisational participatory research study of the feasibility of the behaviour change wheel to support clinical teams implementing new models of care

**DOI:** 10.1186/s12913-019-3885-8

**Published:** 2019-02-04

**Authors:** Eleanor R Bull, Joanne K Hart, Juliette Swift, Kirstie Baxter, Neil McLauchlan, Sophia Joseph, Lucie M T Byrne-Davis

**Affiliations:** 1 Division of Medical Education, University of Manchester, Oxford Road, Manchester, M139PT UK; 2 Department of Psychology, Manchester Metropolitan University, Manchester, M156GX UK; 3 Health Education England working across the North West, 3rd Floor, 3 Piccadilly Place, Manchester, M1 3BN UK

**Keywords:** Organisational change, Behavioural science, Qualitative research, Feasibility studies, Healthcare organisation and delivery, Health services organizations

## Abstract

**Background:**

Health and social care organisations globally are moving towards prevention-focussed community-based, integrated care. The success of this depends on professionals changing practice behaviours. This study explored the feasibility of applying a behavioural science approach to help staff teams from health organisations overcome psychological barriers to change and implement new models of care.

**Methods:**

An Organisational Participatory Research study was conducted with health organisations from North West England, health psychologists and health workforce education commissioners. The Behaviour Change Wheel (BCW) was applied with teams of professionals seeking help to overcome barriers to practice change. A mixed-methods data collection strategy was planned, including qualitative stakeholder interview and focus groups to explore feasibility factors and quantitative pre-post questionnaires and audits measuring team practice and psychological change barriers. Qualitative data were analysed with thematic analysis; pre-post quantitative data were limited and thus analysed descriptively.

**Results:**

Four clinical teams from paediatrics, midwifery, heart failure and older adult mental health specialties in four organisations enrolled, seeking help to move care to the community, deliver preventative healthcare tasks, or become more integrated. Eighty-one managers, medical doctors, nurses, physiotherapists, midwives and other professionals contributed data. Three teams successfully designed a BCW intervention; two implemented and evaluated this. Five feasibility themes emerged from the thematic analysis of qualitative data. Optimising the BCW in an organisational change context meant 1) qualitative over quantitative data collection, 2) making behavioural science attractive, 3) co-development and a behavioural focus, 4) effective ongoing communication and 5) support from engaged leaders. Pre-post quantitative data collected suggested some positive changes in staff practice behaviours and psychological determinants following the intervention.

**Conclusions:**

Behavioural science approaches such as the BCW can be optimised to support teams within health and social care organisations implementing complex new models of care. The efficacy of this approach should now be trialled.

**Electronic supplementary material:**

The online version of this article (10.1186/s12913-019-3885-8) contains supplementary material, which is available to authorized users.

## Background

Globally, health and social care is changing rapidly, with countries making large-scale reorganisations to enable more integrated, person-centred and cost-effective care [1]. In England and Wales, The Wanless Report advocated for prevention and self-management; increased integration between primary, secondary, tertiary and social care; relocation of hospital services to the community; and more home-based care [2]. The Five Year Forward View in England [3] reiterated this, with 50 ‘NHS New Care Model Vanguard Sites’ commissioned in England in 2015, to develop blueprints for new care models [4]. Sustainability and Transformation Partnerships, the next iteration, go even further, urging widespread changes aiming to improve care and deliver efficiency in England [5].

Implementing new models of care is a complex behaviour change intervention [6], requiring commissioners, managers, practitioners, and service users to do things differently [7]. Traditional approaches to implementing policy using regulation and incentives may neglect the vital understanding of individual staff in their context, required to ‘help individuals who work in the NHS make local change happen’ [8], p1. Service transformation at scale requires macro, meso and micro level organisational change to be viewed together, in the context of day-to-day staff practice [9].

Implementation science researchers study health professional behaviour change both by applying classical theories of behaviour to explain health professional practice [10] and by testing theory-based intervention development approaches, including the Behaviour Change Wheel (BCW), synthesised from 18 other behavioural frameworks [11, 12]. The BCW is an approach to behaviour change intervention development centred on three types of psychological determinants required in to enact any behaviour. Understanding these capability (knowledge and skills), opportunity (physical and social environmental barriers) and motivation (both automatic habits and views of pros and cons) barriers to behaviour change for a health professional or group of professionals then enables linkage to different intervention and policy functions to support change (e.g. education, persuasion, guidelines). When designing interventions to overcome barriers, the BCW also recommends specifying specific behaviour change techniques (BCTs) within an intervention [13]. The BCW has been applied to improving sepsis recognition and management [14], diabetes care [15] and energy use [16] and in evaluation of health checks [17], but not yet in organisational changes known as ‘new models of care’.

BCW intervention publications tend to focus more on design than implementation [18] and on content rather than process [19]. Yet for global impact in real-world settings, implementation and evaluation is crucial [20], something well recognised in traditional organisational change theories [21] and in some intervention development approaches. For example, Intervention Mapping [22] more explicitly and clearly defines implementation and the French et al. [23] model emphasises evaluation. Moreover, applying the BCW in large-scale healthcare change may require a flexible research design enabling co-development and refinement with partners. One such healthcare improvement research approach is Organisational Participatory Research (OPR) [24]. OPR emphasises academic and non-academic partners collaborating systematically effect change in an organisation including an ‘iterative research cycle of planning, action and fact finding about the effect of the action’ [25].

Recent evaluations of NHS new care model vanguard sites indicate that implementing organisational change in practice has been a key challenge and that developing shared understandings of local challenges is vital, before testing, evaluating and adapting approaches for continuous improvement [26]. This suggests that a BCW approach with an OPR design may be timely and useful. However, it is unclear both whether the BCW’s focus on health professional behaviour change would be attractive to key stakeholders such as organisational change leaders who may be more macro-level focussed, and how to maximise feasibility and efficacy. This study, taking an OPR approach [24], aimed to explore use of the BCW in implementing new models of care, addressing two research questions:

1. To what extent is the BCW a feasible approach to support teams to change within new models of care?
2. How can we optimise BCW feasibility in this context?

## Methods

### Design

This was an OPR study between academic and non-academic partners. OPR was chosen to enable co-design and refinement of interventions taking into account health system complexities and diversity. Academic partners were health psychologists from the University of Manchester, non-academic partners were Health Education England working across the North West (HEENW), and health and social care NHS new care model vanguard sites in the North West of England. Following OPR practice guidance, a working group was established at study inception with 3 HEE and 3 academic researcher stakeholders, who met regularly throughout to jointly manage the programme [25]. NHS new care model vanguard site leads, and team members were consulted throughout; due to their limited time availability they did not join the working group [27]. Given the focus on healthcare reorganisation and healthcare staff behaviour change, service users were not directly involved in this feasibility study, although would be useful contributors to further work [25].

### Participants / study population

Teams of health and social care professionals from NHS new care model vanguard sites in the North West of England were recruited. We aimed to recruit diverse teams from across the different ‘types’ of NHS new care model vanguard sites including those integrating primary and acute care; moving specialist care to the community; linking hospitals together; joining-up care for older people; and coordinating emergency care [28]. The study and its secondary data analysis met criteria for operational improvement activities exempt from ethical review at the University of Manchester. Participants gave verbal consent to data collection and were assured that they could withdraw from data collection activities at any time.

### Intervention

The study aimed to apply the BCW [14] to help each recruited team to identify key service delivery (behaviour) change required to implement their new model of care, understand barriers and and co-develop an evidence-based, tailored behaviour change intervention to assist implementation. The three stages in the BCW are 1) Understanding the behaviour and determinants, 2) Identifying intervention options (functions and policy categories), and 3) Identifying content and implementation options (including BCTs and mode of delivery).

### Procedure

HEENW sent initial invitation letters to NHS new care model vanguard site leads in the North West of England in May 2016, followed up with email invitations and opportunistic conversations, and meetings with working group members. Interested leads nominated a team from their site having difficulties task shifting, expanding or changing practice to implement new models of care. OPR was thus sought and driven by organisation members directly, an important predictor of success [25]. The BCW was then applied over 18 months, led by the academic partners based on expertise and experience co-developing BCW interventions [29, 30]. Initial results were incorporated into ongoing decision-making and discussions to help optimise applying the BCW [25].

### Planned data collection

A mixed-methods data collection strategy was planned at four key timescales. Exploring feasible data collection methods was part of the study so were not fixed at project inception.

Qualitative methods planned were one-to-one semi-structured interviews with team members, (in person or on telephone), focus groups with team members, written communications, field notes and observations of practice conducted by EB and JS. Semi-structured interview and focus group guides were developed by the working group through literature reviews and discussions with leads and team members and were applied flexibly depending on participant roles, interests and experiences by EB and JS. Interviews and focus groups were audio-recorded where participants consented to this, and audio files were transcribed verbatim anonymously by a professional company and deleted; detailed notes were taken where there were no audio-recordings, anonymous field notes were typed up; qualitative data stored securely.

Quantitative data planned were numeric audit data from observations of practice, questionnaires co-developed with teams to assess staff participant views of the determinants of practice behaviours, based on the COM-B Framework [13] and routinely-collected service evaluation data shared by team leads. COM-B questionnaires, designed using the BCW guide, asked participants to rate their views on physical and psychological capability, social and physical opportunity and automatic and reflective motivation determinants of the behaviour in question, such as for example ‘my colleagues would like me to run a heart failure clinic in the community each week’ for social opportunity. Ratings were using likert scales (1 = strongly disagree, 5 = strongly agree), with some reverse scored to enhance response validity. Specific behavioural determinants were selected from interview data at T1 and T2. Where self-rated behavioural data were collected, participants estimated the number of times they had engaged in the desired behaviour in the previous week. Questionnaires were co-designed and piloted with teams where possible. Quantitative data were entered into a database, with all stored securely on a password protected computer.

### Planned analyses

Qualitative data (transcripts and notes) were thematically analysed using both inductive and deductive analysis across timepoints of the study by SJ and EB. Initial inductive coding relevant to feasibility and efficacy was conducted, a coding schedule agreed, further transcripts analysed separately using Nvivo (v.12) before comparing with recursive discussion and revision of the coding framework. SJ then independently analysed the remaining qualitative data. Theory-led deductive coding was applied by EB to identify capability, opportunity and motivation determinants of practice as part of the BCW process.

We planned to analyse quantitative data by grouping questionnaire scores, quantitative audit data and service level data collected from the various teams to identify common determinants across varied practice behaviours and test for statistical pre-post intervention differences using SPSS (v.22).

## Results

### Participants and intervention focus

**Fig. 1:**
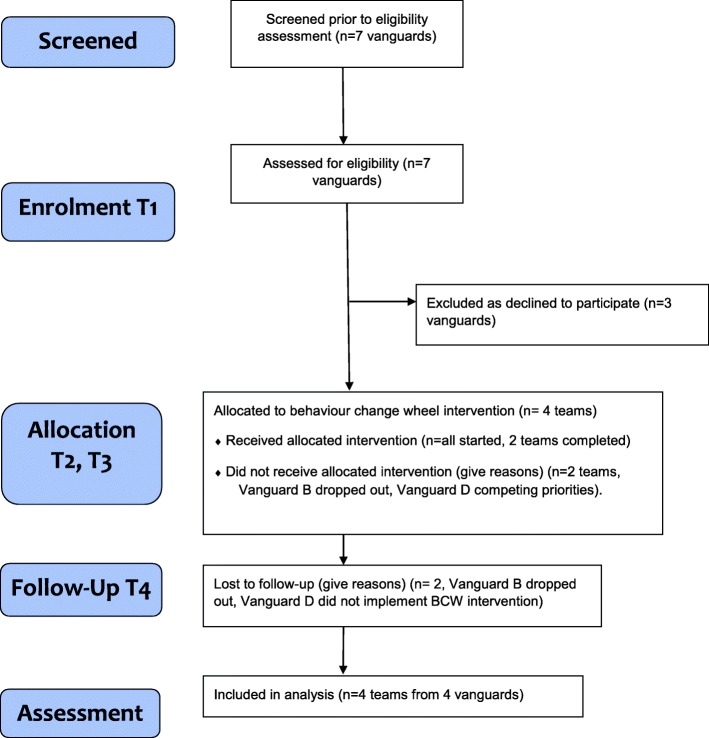
Study consort diagram

Participant flow is included in Fig. 1, and NHS new care model vanguard site and participant characteristics are described in Table 1. In brief, four of seven local sites were enrolled (labelled A-D), collectively serving a population of over three million. We worked with Vanguards A and C to design, implement and evaluate a BCW intervention over 18 months, Vanguard D designed but did not implement a BCW intervention over 12 months, Vanguard B worked with us over six months and did not finish designing a BCW intervention. The four teams of professionals identified covered diverse specialties across the lifespan (paediatrics, midwifery, heart failure, older adult mental health). Eighty-one health professionals contributed data; 89% were women, and there were a wide range of staff cadres (Table 1). Teams chose behaviours related to three types of new care model: integrated working, moving care into the community and expanding preventive roles respectively.

**Table 1:** Vanguard Organisation characteristics

Vanguard	Aim of new model of care	Organisation partners	Local population reach [4]	Team(s)	Health professional participants	Behavioural focus of the intervention
A	Integrated primary and acute care systems vanguard: Multi-disciplinary integrated care	5 partners (incl city council, hospital and mental health trusts, CCGs)	230,000	Integrated health and social care team in an older adult acute mental health unit.	*n* = 36 (29 women, 7 men) 10 Trainee/qualified nurses 6 Vanguard leads/senior managers 6Nursing/activity assistants 3Trainee/qualified physiotherapists 3 Medical doctors 2Trainee/qualified psychologists 2Trainee/qualified occupational therapists 2 Ward managers 1 Speech and language therapist 1 Ward clerk	Increasing the effectiveness of multi-disciplinary integrated working: Ward team instigating more cross-disciplinary recovery-focussed activities with patients.
B	Integrated primary and acute care systems vanguard: Multi-disciplinary integrated care	11 partners (incl NHS trusts, ambulance services, CCGs, local authorities, GP federations)	356,000	Integrated children’s nursing community team	*n* = 10 (9 women, 1 man) 3 Vanguard leads and senior vanguard managers 3 Team leaders 3 children’s specialist nurses 1 service director	Enhancing use of a new integrated service: Increasing referrals from acute staff to a new specialist holistic children’s nursing team
C	Multi-specialty community providers vanguard: Moving specialist care into the community	6 partners (incl local councils, hospital trusts and CCGs)	320,000	Heart failure specialist team.	*n* = 15 (14 women, 1 man) 5 Heart failure specialist nurses (3 acute and 2 community nurses) 2 Administrators 2 Healthcare assistants 2 OD practitioners 1 Vanguard lead 1 Clinical lead doctor 1 Psychologist 1 Physiotherapist	Moving specialist care into the community: Acute heart failure team beginning to run one clinic in the community per week. Community heart failure team redirecting non-specialist referrals back to primary care to increase capacity
D	Acute Care Collaboration Vanguard Site: New care pathways through a network for women and children’s services and engaging more with local helping people to help them better manage their own health.	29 organisations and networks (incl CCGs, hospital providers, and an ambulance service)	Up to 2.4 million	Community midwifery team.	*n* = 20 (20 women) 13 Community midwives 5 Team leads /senior midwives 1 Vanguard lead 1 Clinical lead midwife	Increasing the prevention and self-management role of midwives: Community midwives starting to offer the ‘flu vaccination to every pregnant woman in their care.

### Data collected and analysed

Participants each participated in several types of data collection across the project, described in Table 2. Data collected and analysed were from 50 individual interviews and 11 focus groups or discussion groups held with 57 participant (7 and 3 audio-recorded respectively) observation of 33 team members practicing, a practice audit repeated twice, two sets of questionnaires completed by 20 participants at least once, fieldnotes and two sets of routinely-collected data. All teams contributed data at T1 and T2, A and C to T3 and T4. Quantitative data were analysed descriptively; inferential statistics were not applied given small sample sizes. Questionnaire data collected only at T2 were used solely to target the intervention (see also additional file 1); questionnaire data collected both pre and post (T2-T4) intervention used to evaluate the intervention are presented in the results below.

**Table 2:** Data collection from health professional participants across the study

		*Study time point*															
		T1: Enrolment				T2:Intervention design				T3:Intervention implementation				T4:Post-intervention evaluation			
	*Vanguard*	*A*	*B*	*C*	*D*	*A*	*B*	*C*	*D*	*A*	*B*	*C*	*D*	*A*	*B*	*C*	*D*
Qualitative data	Discussion/focus groups	*n* = 6	*n* = 5			*n* = 14	*n* = 3	*n* = 14	*n* = 19							*n* = 1	
	Individual interviews	*n* = 2	*n *= 2	*n* = 2	*n* = 2	*n* = 27							*n* = 1	*n* = 13			
	Written communication and field notes	5 pieces				40 pieces	22 pieces	26 pieces	12 pieces	10 pieces				15 pieces		4 pieces	
	Observation data					*n* = 32			*n* = 2								
Quantitative data	Questionnaires							*n* = 5	*n* = 15							*n *= 2	
	Audit of team practice					1 audit								1 audit			
	Routinely-collected data							1 set	1 set								

*n *= numbers of staff included where number of participating staff involved were counted

### Intervention delivery and outcomes

The BCW interventions developed with three teams are summarised in Table 3. Interventions designed targeted combinations of C,O and M, through four types of intervention functions and policy categories, and 13 BCTs. Intervention design and delivery with teams A and C are fully described in Additional files 1 and 2.

**Table 3:** Summary of BCW interventions designed with 3 Vanguard teams

	Vanguard A	Vanguard C	Vanguard D
*Summary of intervention focus*	Integrated care in psychiatric ward	Moving heart failure care to community	Midwives offering preventive ‘flu vaccine
*Relevant psychological determinants identified (C, O or M)*	C, O and M	Community sub-team: O Acute sub-team: O and M	C and O
*Intervention functions proposed*	Persuasion Environmental restructuring Training	Enablement Environmental restructuring Persuasion	Training Environmental restructuring
*Relevant policy categories*	Environmental/social planning Service provision Guidelines	Communication/marketing Environmental/social planning Service provision	Service provision Environmental/social planning
*BCTs proposed (from* [12]*)*	1. Information about health consequences 2. Information about social and environmental consequences 3. Instruction on how to perform behaviour 4. Demonstration of behaviour 5. Action planning 6. Restructuring physical environment 7. Restructuring the social environment 8. Reviewing behavioural goals	*Community sub-team:* 1. Behavioural substitution 2. Social support (practical) 3. Adding objects to the environment *Acute sub-team:* 4. Feedback on behaviour 5. Behavioural experiment. 6. Action planning 7. Environmental restructuring	1. Adding objects to the environment 2. Demonstration of behaviour 3. Instruction of how to perform the behaviour

In the seven-day audit of rehabilitation activities in Vanguard A, 17 activities were held pre-intervention, 18 post-intervention. ‘Types’ of activity leader involved doubled, from 4 pre-intervention to 8 post-intervention, including patients In Vanguard C, the limited pre-post questionnaire data collected from acute nurses (*n* = 2) suggested that for C and O determinants, plans and expectation to run clinics in community increased post-intervention, with scores on the 5-point ‘plans’ scale doubling (mean 1.67 to mean 3.5;see Additional file 2).

### Feasibility themes

The inductive thematic analysis of qualitative data revealed 5 main themes identifying to what extent the BCW was a feasible approach for this context and how to optimise this. These were:

*Qualitative data is most feasible, Making behavioural science attractive, Key mediators: Co-development and a behavioural focus, Ongoing communication with teams Support from engaged leaders,* discussed below.

#### Qualitative data is most feasible

Analysis of field notes from initial meetings and communications revealed that team leads felt qualitative data most feasible to collect. Some reported this would be most efficacious, giving deeper insights into barriers and facilitators of practice change, others that it would engage team members in the project through providing a listening space, others that it would help build rapport. Equally in this theme, field notes suggested managers feared that asking teams to complete quantitative COM-B questionnaires would make them feel ‘tested’ or watched, disengaging them. Additionally, it was noted that service-level data was not easily shared with the working group, because of reservations about data protection; quantitative observation and audit were revealed as most acceptable forms of quantitative data collection.

#### Making behavioural science attractive

This theme included interviews and field notes, where it was noted that engaging Vanguards in the BCW was challenging, beginning with reaching decision makers in hugely complex, multi-layered organisations. Concerted, repeated contacts with many layers of health service management were required. For Vanguards deciding not to participate, written communications suggested they did not see the relevance of the programme, for instance one Vanguard lead from a non-participating vanguard optimistically expressed:

‘*I don’t think we need this programme as our teams are all fully integrated’* Vanguard lead, written communication from HEENW.

Another Vanguard lead was concerned about perception of behavioural science and of time demands in engaging teams:

*‘It will be a real help, we just need to persuade them that’s what they need and it’s not management mumbo-jumbo: clinicians have a natural distrust for behavioural methodology … also a ‘phone chat’ may be more appealing than calling it interviews’* Vanguard B lead, T1, individual interview.

Attractive and informative communications about the programme were required, a ‘reflective loop’ taken by the working group to optimise the programme. The brand ‘Teams Together’, a colourful logo, short PowerPoint presentation and animation about the BCW developed by the programme team (available at http://www.mcrimpsci.org/elearning/) at T1 to communicate the programme effectively. In another reflective loop, a Vanguard lead advised including more evidence about BCW to engage clinical colleauges. Fieldnote data suggested this optimised the attractiveness of the BCW, for instance:

‘*These materials are very helpful for homing in and clarifying..we can pair the programme with an existing innovation and project’.* Vanguard C Lead, T1, individual interview.

Within this theme, another aspect documented in interviews and field notes concerned explaining the BCW to non-psychologist colleagues. Reflexive fieldnotes from initial meetings suggested behaviour change terminology used in the BCW should be simplified, and intervention implementation and evaluation emphasised to optimise engagement in a BCW intervention in this context. For instance one senior manager in Vanguard A commented:

‘*So this behaviour change wheel helps you make a programme to help the staff but what is going to happen and who is going to actually do it and evaluate it – is there support? We’d worry about sustainability.’* Vanguard A senior manager, T1, focus group meeting.

**Fig. 2:**
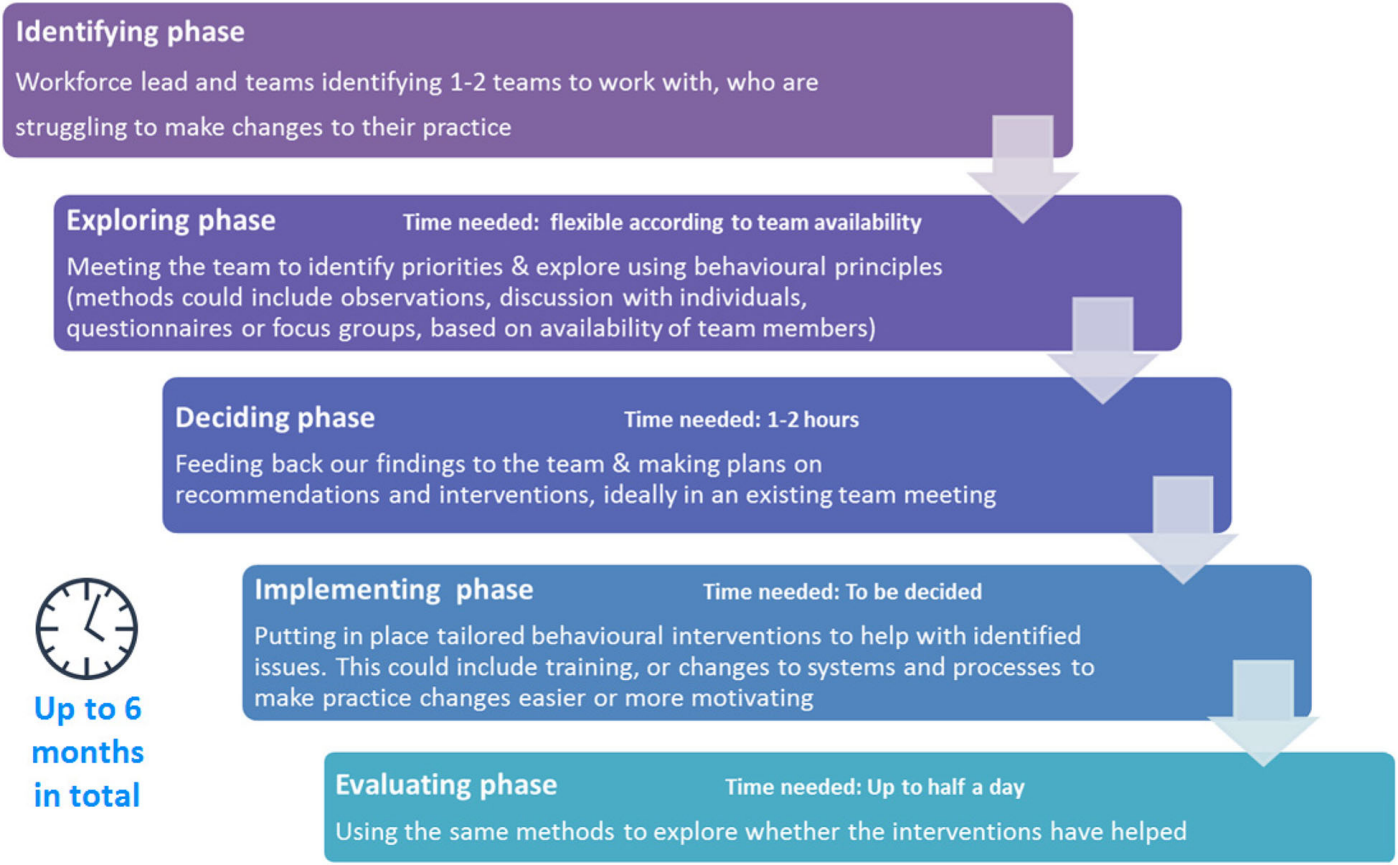
Five stage process of the Teams Together Programme

To optimise feasibility, as a key reflective loop, we developed a five-phase process to implement the BCW in this context, summarised in Fig. 2. Stage 1 of the BCW corresponds to the identifying and exploring phases, stage 2 and 3 are contained within the deciding phase.

#### Key mediators: Co-development and a behavioural focus

A third feasibility theme concerned how the intervention was perceived to work. T4 evaluation data suggested that co-development and collaboration as well as a behavioural focus had been essential ingredients, or mediators of changes in capability, opportunity and motivation determinants and behaviours. The motivation and confidence-building process of staff becoming actively involved in change, through the BCW and facilitated by the OPR approach was seen as vital to the success of the programme, as discussed by a ward manager:

*‘It was so important that the staff wanted to focus on activities and rehab …*. *it came from the staff and the away days in particular helped bring out the importance of recovery for people and get their own ideas out for how to change things … it sparked new ideas that we could work on. Now everyone is spurred up and their ideas are brilliant’* Vanguard A ward manager, T4, individual interview.

Co-development also meant that tailoring was possible, for timescales, BCW content and delivery methods. Team members themselves were encouraged to deliver BCTs from the behaviour change wheel, such as the team champion delivering rehabilitation coaching. It was important to consider who could be the deliverers of interventions especially those aiming to build motivation, to be a ‘credible source’ for teams.

Taking a behavioural approach was generally appreciated by frontline staff who felt this operationalised and personalised new care models, as in an interview in T4:

*“They* [away days] *were definitely beneficial, no doubt about it, it gave some structure and a forum in which to say that that is what everybody wants to do, everybody has got their own things that they think should be happening and it gave everybody a place to voice their ideas.”* Assistant Occupational Therapist, Vanguard A, T4, individual interview.

Equally, managers felt that this approach helped specify the changes, such as the clinical lead in vanguard C:

*“Teams Together helped me define what we have to do into just two or three changes”* Clinical lead, Vanguard C, T3, written communication.

However, teams and vanguard leads varied in how easy they found specifying a behaviour. Field note analyses suggested that teams who had a clearer idea of what changes may mean for their pathway were more ‘ready’ for the approach and could immediately see its relevance, and some wanted help with ‘decision making’ which is not overtly behavioural.

‘*It is more about the decision making, we all know what we need to do but it’s how to get there that is a problem’.* Vanguard Lead B, T1, individual interview.

All teams focussed on behaviours they wished to ‘start’ doing or do more of rather than ‘stop’ doing or do less of.

#### Ongoing communication with teams

Across timepoints, analysis of fieldnotes particularly emphasised the importance of regular communication when taking an OPR approach, where weeks could pass without face-to-face meetings with team members. Interestingly, feeding back to teams, which is an essential aspect of OPR, was most feasible with a ‘low-tech’ approach to this feedback, using flip charts and post-it notes, and displays around the wards. Interviewees reported this helped them feel they could contribute to ideas and the results were not already finalised. Highlighting positive behaviours and team strengths observed during feeding back also helped engage teams. Paper-based solutions were also useful in Vanguard C’s behavioural experiment: the patient survey had been originally conceived on an ipad, but after delays and technical difficulties, there was more success with a simple printed sheet, which nurses managed to distribute rapidly to numerous patients.

In vanguard A, the first to reach the deciding phase, a report was disseminated by email and copies left on the ward, as well as a PowerPoint presentation, delivered at the first away day and distributed via email. Informal enquiries suggested few team members had read even the executive summary of this report, despite the report being heavily pictorial and colourful. In work with the later teams, colourful one-page summaries seemed a more acceptable means of communication about BCW interventions happening in teams.

#### Support from engaged leaders

Finally, a vital recurring feasibility theme from qualitative data was support from engaged leaders, crucial to embed and champion the programme. In Vanguard B, behaviours were difficult to identify since several team leaders involved had conflicting ideas about new models of care aims and implementation. Field note analysis reflected on the difficulty that in this Vanguard, a key team leader appeared to have low motivation for the behaviour change chosen by others, therefore unsurprisingly did not offer support for a BCW intervention to help their team implement it, a factor influencing the difficulties maintaining Vanguard B's engagement in the programme.

Furthermore some leaders who were themselves in favour of the change but suspected their teams to have motivational barriers, initially felt that engaging in the programme could be too strong a personal endorsement of the change, causing relationship problems with teams. In Vanguard A, the T4 interview with a manager yielded the invaluable insight that investing time and public support in a BCW could at first feel risky:

*“It was quite overwhelming when you first came.. it was just me … .my thoughts about what they might say to you, whether it would be all negative when you spoke to them, whether it would work and they would be better or if it wouldn’t work and things would get worse. But in fact it’s been the opposite, it’s all been really positive”* Vanguard A manager, T4, individual interview.

Reflective field notes compared this with the co-development approach taken by team C’s leader, a clinician who decided to present the team with the clinical evidence suggesting the need to change, and enabled them to draw their own conclusions about what behaviour changes could be needed, which she then restated as options.

Internal support and leadership was also seen as important to sustaining a BCW intervention. In interviews with frontline staff from vanguard A at T4, several team members were concerned about ongoing sustainability where the team leader had encouraged the staff group and the academic partner to lead the intervention. One health care assistant commented:

*‘There has not been a leader … .someone more senior who would have led it and chased it up and that … .someone who is here all the time … chasing it up, making sure it is being done daily until everybody gets into that rhythm’.* Healthcare assistant, Vanguard A, T4, individual interview.

Internal leadership may also have shielded the programme from competing demands which delayed planned interventions in vanguards C and D. In an interview with vanguard lead D when the planned intervention implementation had been on hold for several months, she suggested that time pressures were to blame:

*“I mean I think they were actually really keen for you to do the, from a midwifery perspective, that follow-up training you were going to do with them. It has definitely been the added time pressure constraints … they’ve just been unlucky in terms of a number of things that have factored in with extra training they had to do these last few months, a lot of pressure”* Vanguard D lead*,* T3, individual interview.

Nevertheless, the team from Vanguard A almost unanimously agreed that BCW development needed to be led by an ‘outsider’ who they felt offered a fresh perspective, an unbiased view of team dynamics and a behavioural scientist’s skills:

“*I think it’s good to have a psychologist taking on that role. I think just because you can see things from different angles and understand … being able to engage with people and make them feel comfortable to talk to [ …*] *having a wider understanding I think of teams and team dynamics is useful*.” Nurse vanguard A, T4, individual interview.

## Discussion

In an OPR study, academic and non-academic partners developed and implemented BCW-based health professional change interventions with teams from NHS England Vanguards implementing new models of care. We identified behaviours and explored barriers with four teams, developed interventions with three teams; implemented and evaluated interventions with two teams, collecting qualitative and some quantitative data to explore how to optimise feasibility. Five feasibility themes emerged from analysis of the qualitative data collected, emphasising the importance of engaging the right health service partners from complex and dynamic organisations through co-development and effective communication about behavioural science.

One feasibility theme summarised participants’ view that the behavioural approach to interventions was a key mediator of change. The selection of behaviours in a BCW intervention is not always straight forward and health professionals may struggle to define and prioritise behaviours, an area where psychologists may play a useful role [31]. Yet, in most behaviour changes (aside from environmental changes automatically cueing new behaviours) people must be aware of what behaviours to change: our findings illustrate that staff did not know what the new model of care meant they should do differently and this would certainly lead to a lack of change. We also found that behaviours chosen for the BCW intervention tended to be those teams wanted to start or do more of, not less of or stop. This may be because these were most easy for teams to conceptualise. Behaviours vary from each other in several key dimensions and at present behavioural science has no typology of behaviours to categorise them [32, 33]. Further scientific work in this area would help define and study which may be most appropriate for a BCW intervention in this context.

Additionally the finding that co-development was an important perceived mediator of change and aspect of feasibility has implications for policy makers, organisational change practitioners and managers involved in service redesign. Co-development is a key principle of the OPR approach itself [24], but also resonates with the organisational change literature, where there is increasing emphasis on co-development and co-creation of interventions [34], aimed at ensuring sustaining change and an engaged workforce. The NHS Constitution also pledged that staff would be empowered to drive changes in their health and social care organisations [35]. However, our feasibility themes suggested that this was a unique aspect of the programme uncommon in staff’s experiences of implementing new models of care. Indeed, other exploratory work we conducted, discussed elsewhere, found evidence that staff perceive top-down, prescribed change in new models of care as being both a cause and consequence of poor workplace culture for teams [36]. Health psychology approaches such as the BCW can be a tool to put change back in the hands of the team, supporting self-efficacy and this was appealing to managers keen to implement change whilst wary of putting extra demands on teams. However, our study also underlined the importance of the methods and principles of application. The feasibility data emphasised that the BCW must be applied in partnership *with* teams rather than to conduct a study *on* them, which builds on our understanding about using behavioural science in practice. Policy makers and managers may find that co-designed and implemented, behaviourally-focussed interventions may help the NHS deliver on some of the aims of the NHS Constitution and improve staff work engagement. Organisational change practitioners may also benefit from training adding the BCW or other behavioural approaches to their toolbox.

The feasibility themes highlighted engagement, time and leadership as important issues. It seems ironic that teams could be too busy struggling with changing to benefit from support to make the change, but others note the time and effort needed from motivated individuals to begin to spark intra-organisational health partnerships [37]. As we expected, an important part of optimising feasibility involved implementation and evaluation, including extending the BCW’s three stage intervention development process. It may also be that engagement and ownership would be further increased by having team representatives, if not clinical leads, as well as service users, at working group meetings.

This study has several limitations, not least that its flexible design came at the cost of not collecting substantial quantitative data. We focussed only on teams’ practice behaviour and psychological determinants, without measuring service-level indicators of team performance and successful implementation of new models of care such as staff sickness and turnover, staff engagement or burnout. These would be important indicators in a future feasibility trial. The four teams we worked with, although diverse and representing a range of new models of care, were local to North West England. Further work is needed both to collect efficacy data and sample from a wider range of teams.

## Conclusions

Overall, this OPR study to explore BCW feasibility suggests that BCW may be a helpful approach for teams who are the ultimate pathway in implementing large-scale organisational change. Attention to the process of developing and implementing implementation science interventions is important, with feasibility maximised through co-development, strong leadership and effective communication about behavioural science.

## Additional files

Additional file 1: Vanguard A Case Study (.doc file) case study illustrating application of the BCW with the team in Vanguard A (DOCX 16 kb)

Additional file 2: Vanguard C Case Study (.doc file) case study illustrating application of the BCW with the team in Vanguard C (DOCX 15 kb)

## Abbreviations

BCT: Behaviour Change Technique
BCW: Behaviour Change Wheel
HEE: Health Education England
OPR: Organisational Participatory Research
