# Supplementary material for: An organisational participatory research study of the feasibility of the behaviour change wheel to support clinical teams implementing new models of care
Source: BMC Health Serv Res. 2019 Feb 4;19:97. doi: 10.1186/s12913-019-3885-8 (PMC6360652; doi:10.1186/s12913-019-3885-8)
Supplement: Supplementary file 1 — Vanguard A Case Study (.doc file) case study illustrating application of the BCW with the team in Vanguard A (DOCX 16 kb) [file 12913_2019_3885_MOESM1_ESM.docx]

**Additional file 1: Vanguard A Case Study**

| **VANGUARD A CASE STUDY**  The team from vanguard A was an acute older adult mental health team working in a new 18 bed community residential unit. The team was fully integrated, including a wide range of health and social care staff with visiting social workers. The team had formed 18 months previously with new members, and approximately half having been moved from an acute facility in a neighbouring town as part of the vanguard’s reorganisation of care to be more integrated and community-based. Senior staff in initial interviews suggested the team was *‘change fatigued’*, levels of sickness and staff turnover relatively high, and that morale, relationships amongst staff and with managers were strained.  We agreed that to engage with the team in the exploring phase, a qualitative approach was preferable, including a period of observation and individual interviews, focussing on gathering staff’s own ideas for change in contrast to change imposed upon them. The team were informed of the programme via a welcoming poster and email communications and stage 1 of the Behaviour Change Wheel was completed through over 90 hours of participant observation and short interviews by EB and JS. Amongst six possible behaviours discussed with the team, they chose to work on *people instigating more cross-disciplinary recovery-focussed activities with patients*, something that they felt should be a shared goal across their new integrated team. An audit conducted by the assistant occupational therapist established baseline numbers and types of recovery-focussed activities.  Analysis of observation field notes and 32 interviews suggested that capability, opportunity and motivation were all relevant determinants of practice. Feeding back options to the team and a wider steering group, we agreed that training, persuasion and environmental restructuring were most relevant intervention functions of the behaviour change wheel. These were delivered over 4 months focussed on two team away afternoons with an intervention period in between. The main behaviour change techniques were:   - **Information about health consequences and about social and environmental consequences:** Reflective exercises and presentations on the benefits of rehabilitation activities for patients and assurance that this was part of all team members’ roles. Delivered at the first away day by allied health professionals to increase team *motivation*. - **Instruction on how to perform behaviour:** Informal education and a resource pack of activity ideas developed by nursing assistants who were rehabilitation champions, to increase team *capability*, in the period between away days. - **Demonstration of behaviour:** Offers by nursing assistants for less confident members of staff to shadow them in leading rehabilitation activities over the period between away days, and how to encourage patients to lead activities to increase team *capability* for rehabilitation on the ward. - **Action planning:** Team activity in both away days, facilitated by EB and JS, to support the team to agree on and specifiy what changes would be made, by whom, when and how. This helped team members make use of *social and physical opportunity* for change. Sub groups for each theme of work were developed to help take the programme forward. - **Restructuring physical and social environment:** Physical changes to the ward were agreed and implemented largely by the ward manager. This included changes to location of family visits to promote social cohesiveness in the main lounge area, creation of an activities room, introduction of a darts and pool table for patients and efforts to link with charities and organisations who could organise rehabilitative activities for patients. - **Reviewing behavioural goals:** Review of these activities in the second away day.   Reauditing behaviours following the intervention suggested that whilst absolute numbers of activities taking place on the ward per week was not substantially different (17 vs. 18), the ‘types’ of staff involved in this had doubled (4 to 8) following the programme, including patient-led activities. The occupational therapist assistant also reported that it was becoming very difficult to audit activity numbers, since in his view informal , short recovery-focussed conversations and activities were appearing ‘*left, right and centre’*.  The month following this, the ward manager reported that staffing levels were back almost to their full complement with fewer people on sick leave. In 13 interviews, team members reflected on progress and the impact of the programme. Participants frequently commented that morale had improved after the initial difficulties, and that practice behaviours had changed on the ward in the past months:  “*When [new staff] moved here it, kind of, just upset everything and it was, like, starting again really, building relationships* […] [the team] *had a bit of a change of dynamic really*.” (Participant 3)  “*The staff work really well on this ward and there’s been a big improvement*.” (Participant 1)  “*I’ve noticed more the nursing team running more Therapeutic Groups on the ward, I think. I think they’re…a lot of them are quite keen to get involved in that and maybe understand the benefits of that in terms of recovery a bit more after the Away Day that we had*.” (Participant 5)  *‘The staff are doing the job more with the activities …they’re doing something now that they weren’t necessarily doing a few months ago. ..there’s a feeling on the ward now …more togetherness between people and more of a laugh. Yeah, all that coming together…from the away days I think’ (Participant 3)*  Team members also felt the focus on rehabilitation had been useful in terms of having a shared goal to achieve and that this would become intrinsically motivating:  “*When staff have an activity they can see someone getting involved who doesn’t usually take part, see that little spark coming back in their eyes when they take part and the staff know they played a part in it, and eventually they’ll get to go home and the staff know they helped in the recovery of that person, and that is really inspiring for them because they can see the effects of what they’re doing by encouraging them to take part in things. And that will inspire them to do even more, I think*.” (Participant 2)  However, staff members also reported ongoing challenges, particularly identifying the need for a leader within the staff team to head the programme for the staff and ensure Teams Together tasks were being implemented as agreed. |
| --- |
