# Supplementary material for: An organisational participatory research study of the feasibility of the behaviour change wheel to support clinical teams implementing new models of care
Source: BMC Health Serv Res. 2019 Feb 4;19:97. doi: 10.1186/s12913-019-3885-8 (PMC6360652; doi:10.1186/s12913-019-3885-8)
Supplement: Supplementary file 2 — Vanguard C Case Study (.doc file) case study illustrating application of the BCW with the team in Vanguard C (DOCX 15 kb) [file 12913_2019_3885_MOESM2_ESM.docx]

**Additional file 2: Vanguard C Case Study**

| **VANGUARD C CASE STUDY**  The team from Vanguard C was a specialist heart failure team, containing specialist nurses, consultant cardiologists, a physiotherapist, psychologist and adminstrators and led by a consultant cardiologist. The focus of change was particularly for the acute and community sides of the nursing team, who had previously worked separately. The team lead aimed to move towards a new model of care of greater community based care.  Initial meetings with the team lead revolved around defining the problem in behavioural terms and selecting and specifying behaviour, a process the consultant cardiologist commented ‘*helped me define what we have to do in just two or three changes – invaluable’* . We realised that for that team, moving care to the community meant streamlining the community-based nurses’ activities in primary to free-up capacity, and acute staff beginning to run some clinics in the community. Key behaviour changes were identified as the acute heart failure team *beginning to run one clinic in the community per week*; the community heart failure team *redirecting non-specialist referrals back to primary care to increase capacity*. The programme was run alongside another initiative to create and implement a joined up IT system between the two teams.  As part of the exploring phase, a COM-B questionnaire delivered as part of a wider team learning day, suggested the community staff capability and motivation to redirect inappropriate referrals were high, but there were social opportunity barriers (perceived social pressure from practice GP and nurses). For acute staff, capability to deliver in the community was high, but motivation and opportunity were lower, with particular worries that patients wouldn’t like the changes. In the deciding phase, we agreed that the functions of enablement, environmental restructuring and persuasion were most relevant and planned some activities incorporating several main behaviour change techniques:   - **Behavioural substitution, Social support (practical)** and **adding objects to the environment:** Community staff to develop a feedback tool to accompany re-referrals back to primary care staff with reassuring information about the range of other services available for them - **Feedback on behaviour:** Using service-level data to shed more light for the community team about how often this practice was happening already (there were varied views) - **Behavioural experiment:** For the acute team to conduct a behavioural experiment survey on patient care preferences to test out their worry that patients wouldn’t like clinics to be run in the community. - **Action planning** and **environmental restructuring:** To overcome identified physical opportunity barriers to running clinics in the community.   Following this, a period of delay occurred whilst the team struggled to implement the joined-up IT system initiative. We however did manage to stay in touch with the acute team who wished to proceed with the patient preferences behavioural experiment. We co-developed the survey around where and with whom patients would prefer to receive their follow-up care post-discharge from hospital. Staff surveyed 48 heart failure patients; we helped analyse this anonymous data and presented back the findings to 2 of the 3 acute nurses and service lead in a PowerPoint presentation and one page summary. The findings suggested that in contrast to their fear that 100% would prefer to be seen back in hospital, amongst those surveyed the figure was closer to 37% with 44% preferring a community venue.  At this meeting, we also discussed opportunity barriers and implemented action planning techniques. The two nursing team members re-completed the COM-B questionnaire and team lead was interviewed. In comparing the very small sample descriptively, ratings on capability and opportunity determinants had slightly increased, and expectation to run a clinic in the community had increased (mean 2.5 – mean 4/5) scores for plans of ‘how, when and where they could run a clinic in the community each week’ had more than doubled (mean 1.67 – mean3.50/5). Free text comments suggested enhanced motivation, such as ‘I see the need for clinics to be developed in community in addition to acute hosptial settings’. Next steps for the programme were to embark on the opportunity barriers through the period of shadowing other professionals and environmental resturcturing. The team leader felt confident she could help the team work towards these next steps independently.  The team leader commented about the programme ‘*Your advice and support is very much appreciated. I have no doubt that the approach has significantly helped us engage the work force in change. I hope we have opportunity to work with you again in the future’.*, and that ‘*the team are more engaged, less overwhelmed, actively exploring how to put changes into practice’.* |
| --- |
